# Carboxytherapy for Genitourinary Syndrome of Menopause: A Pilot Study

**DOI:** 10.1093/asjof/ojag019

**Published:** 2026-02-16

**Authors:** Antonella De Ponte, Sara Sofía Bermúdez Sparice, Luciana Bergamaschi, Sonia Baulies, Sandra Garcia, Ignacio Rodríguez, Silvia Cabrera

## Abstract

Carboxytherapy has emerged as a promising nonhormonal therapy for genitourinary syndrome of menopause (GSM) and associated female sexual dysfunction. GSM, characterized by vulvovaginal tissue changes because of estrogen depletion, often leads to vaginal dryness, reduced elasticity, and dyspareunia. This pilot study aimed to evaluate the preliminary efficacy and safety of carboxytherapy in postmenopausal women with GSM. In this prospective pilot trial, postmenopausal women with GSM underwent 5 sessions of controlled CO_2_ administration using a specialized carboxytherapy device. Outcomes were assessed using the Bachmann Vaginal Health Index (VHI), the Vulvar Health Index (VuHI), the Female Sexual Function Index-6 (FSFI-6), and pain measured with the visual analog scale (VAS). Evaluations were performed at baseline and 1 month after treatment. Nineteen women completed the study. Posttreatment assessments demonstrated significant improvements in VHI scores (from 12.55 ± 2.56 to 18.26 ± 3.72; *P* < .001) and decreases in VuHI scores (from 9.80 ± 2.40 to 3.37 ± 3.67; *P* < .001). FSFI-6 scores improved from 12.55 ± 4.70 to 17.95 ± 7.61 (*P* = .017). Pain during treatment was minimal, with a mean VAS score of 1.95 ± 0.94. Carboxytherapy appears to be a safe, well-tolerated, and potentially effective nonhormonal option for managing GSM, particularly in cases with significant vulvar involvement. However, given the small sample size, lack of a control group, and short follow-up, these findings should be considered preliminary. Larger randomized controlled trials with longer follow-up are needed to confirm the durability and generalizability of these results.

**Level of Evidence**: 4 (Therapeutic)

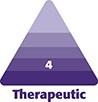

The genitourinary syndrome of menopause (GSM) is characterized by anatomical and functional changes in vulvovaginal, urethral, and bladder tissues because of estrogen depletion. Symptoms include vaginal dryness, burning, decreased lubrication, dyspareunia, urinary urgency, and recurrent urinary infections.^1^ The prevalence of GSM is estimated at ∼50% among postmenopausal women, increasing with age.^2^ When diagnosed based on symptomatology, the prevalence can exceed 70%, and in cases with at least 1 vaginal symptom, the diagnosis is confirmed in over 90% of cases.^3^ Many women with GSM present with sexual dysfunctions, especially pain-related disorders.^4^

Topical estrogen therapy remains the gold standard for GSM, because it alleviates symptoms by restoring local estrogen levels.^5^ However, certain patients are unsuitable candidates for hormonal therapy, including those with unexplained vaginal or uterine bleeding, estrogen-dependent malignancies, a history of thromboembolic events, or estrogen sensitivity.^6^ Others prefer to avoid hormones altogether. Additionally, some individuals only experience partial relief from local hormonal therapy. As a result, there is an increasing demand for nonhormonal, regenerative options, among which carboxytherapy shows potential.

Considered a safe and low cost procedure, carboxytherapy consists of controlled insufflation of carbon dioxide (CO_2_) into the subcutaneous tissue.^7^ The increase in the local concentration of CO_2_ triggers a reaction with the water contained in the tissue, which lowers the local pH and enhances the Bohr effect, thereby facilitating oxygen dissociation from hemoglobin and improving tissue oxygenation.^8^ Additionally, it promotes the transport of proteins necessary for extracellular matrix remodeling and tissue regeneration.^9,10^

Some studies suggest that vasodilation induced by carboxytherapy could be mediated by nitric oxide, enhancing the neoangiogenic properties of CO_2_ and inducing the expression of angiogenic factors, such as vascular endothelial growth factor and growth factor of fibroblasts, as well as the inhibition of endothelial cell apoptosis. The use of CO_2_ in angiographic procedures has demonstrated the safety of this gas, which is both economical and nonallergenic.^11^ Recent research has positioned carboxytherapy as a safe and economical technique for various pathologies in vascular, aesthetic, traumatological, dermatological, and urological areas.^12,13^ In preliminary studies, carboxytherapy has demonstrated potential usefulness in gynecological pathologies.^14^

The objective of this research was to evaluate the impact of carboxytherapy in the treatment of GSM, as well as to assess treatment tolerance and the occurrence of side effects.

## METHODS

### Study Design and Population

**Figure 1.**
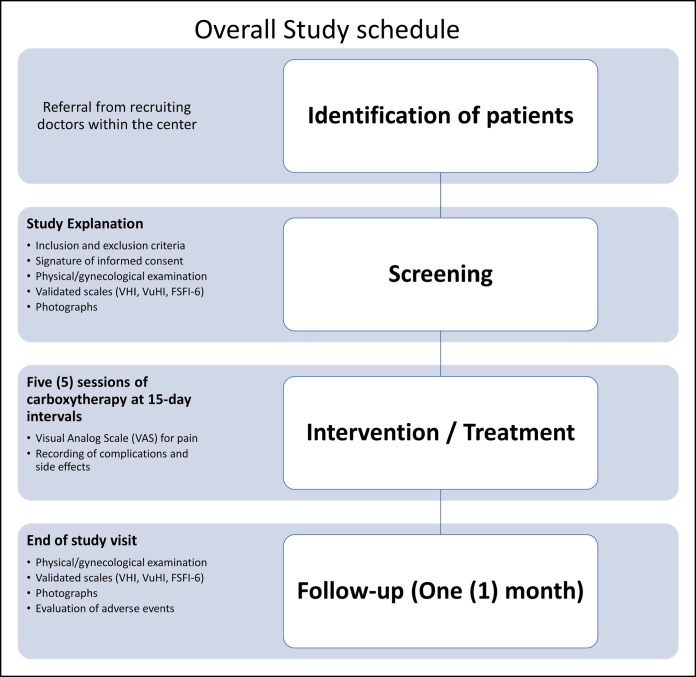
Study schedule.

Patients were consecutively recruited from referrals within our institution during the study period (2021-2023). All women presenting with symptoms of GSM were screened according to predefined inclusion and exclusion criteria. Eligible participants received a detailed explanation of the study procedures, treatment, and follow-up schedule, and those who agreed signed written informed consent before baseline physical and gynecological examination, completion of validated scales the Bachmann Vaginal Health Index (VHI), the Vulvar Health Index (VuHI), the Female Sexual Function Index-6 (FSFI-6), and standardized photographs (see Study Schedule in Figure 1). All screening and follow-up gynecological examinations, as well as the carboxytherapy procedures, were performed by 2 senior investigators, both specialist physicians in Obstetrics and Gynecology from our unit.

Twenty postmenopausal women diagnosed with GSM were enrolled at our institution between 2021 and 2023. Inclusion criteria included postmenopausal women under 75 years of age who were sexually active and had not received systemic or local estrogen therapy within the previous 3 months. A Bachmann VHI score below 15 was required for inclusion. Women with connective tissue diseases, recent hormone therapy, vulvar pathologies, or contraindications for carboxytherapy, including renal, cardiac, or pulmonary insufficiency and active infections were excluded.

### Treatment Protocol

Participants underwent 5 sessions of carboxytherapy at 15-day intervals. CO_2_ was administered subcutaneously with a Dioxage device Skymedic, CE 0476, manufactured in Spain, calibrated to regulate CO_2_ dose, a flow of 25 mL/min, and prewarming to enhance comfort.

Patients were placed in the gynecologic lithotomy position. After removal of the topical anesthetic, the vulvar area was cleansed with chlorhexidine before the injections (Video). Multiple micro-infiltrations with CO_2_ were performed until a soft, self-resolving subcutaneous emphysema was achieved, mainly in the labia minora, clitoral hood, vaginal introitus, and lower third of the vaginal mucosa, avoiding the urethral meatus. The number of injection points and the distribution of CO_2_ were adapted to individual anatomy and tissue response. The acute tissue response to CO_2_ infiltration is characterized by transient subcutaneous emphysema and vasodilation, which resolve spontaneously within minutes and trigger improved local perfusion and oxygenation. The maximum CO_2_ dose was 2500 mL per session, delivered with a 30 gauge needle at a depth of 1 to 4 mm and a 10° angle. To reduce discomfort, 3 mL of Emla cream (25 mg/g of lidocaine and 25 mg/g of prilocaine, Aspen Pharma Trading Limited, Ireland) was applied to the treatment area with occlusion for 20 min before each session. The use of prewarmed CO_2_ at low flow rates, together with topical anesthesia, was part of the protocol and aimed to optimize patient comfort during each treatment session. Complications and adverse effects were systematically monitored and recorded at every session.

### Assessment Tools and Outcome Measures

Primary outcomes were assessed using the Bachmann VHI, which is a scale that evaluates 5 key aspects of vaginal health: elasticity, secretions, pH, mucosal state, and hydration.^15^ A low score indicates deterioration in vaginal health, with values <15 associated with vaginal atrophy. The VuHI quantitatively assesses vulvar characteristics, evaluating 5 aspects: labia majora and minora, clitoris, vestibule, pain, and elasticity; a low score suggests good vulvar status.^16,17^

Sexual function was assessed by the FSFI-6. The FSFI-6 is a validated questionnaire with sensitivity and specificity >94% in identifying sexual dysfunction, as demonstrated in validation studies (*P* < .001), is suitable for use in daily clinical practice, assessing desire, arousal, lubrication, orgasm, satisfaction, and pain during sexual intercourse. A score ≤19, or when the score of any domain is <3.6, is considered indicative of risk of sexual dysfunction, whereas a value >19 is associated with normal female sexual function.^18-20^

Symptoms reported by patients were dryness, itching when cleaning, dysuria, urinary urgency, pruritus, dyspareunia, and trigger point pain (6 o'clock). Pain was assessed using a visual analog scale (VAS), and standardized photographs were taken at baseline and 1 month after treatment and were evaluated by 2 gynecologists from our unit, both of whom contributed as coauthors to this study and were not involved in patient screening or treatment, based on their clinical judgment of vulvar appearance. Adverse events were evaluated at every follow-up visit.

### Statistical Analysis

Descriptive statistics were performed by analyzing the mean and standard deviation. Wilcoxon test for paired samples was used to compare scales between pre- and posttreatment. All tests were 2-tailed, and *P* < .05 was considered statistically significant. All analyses were performed using IBM SPSS Statistics v29 (Armonk, NY).

### Ethical Considerations

The study was approved by Ethics Committee of the Grupo Quirónsalud-Catalunya, approval number 12/2021, and all participants provided written informed consent. The study followed the principles of the Declaration of Helsinki.

## RESULTS

Of the 20 patients recruited, 19 completed the 5 treatment sessions and the follow-up assessment 1-month posttreatment.

Significant improvements were observed in VHI. The mean VHI score increased from 12.55 ± 2.56 pretreatment to 18.26 ± 3.72 posttreatment (*P* < .001), indicating an improvement in vaginal health. Table 1 presents the results of each VHI parameter before and after treatment. The VuHI scores decreased from 9.8 ± 2.40 pretreatment to 3.37 ± 3.67 posttreatment (*P* < .001), indicating an improvement in vulvar health. When analyzing the individual domains of this scale separately, we observed improvement at all levels, especially in pain decrease, elasticity improvement, and labia majora and minora anatomy characteristics (Table 2).

**Table 1.** Improvement in Parameters of Vaginal Health Index Pre- and Posttreatment

	Pretreatment		Posttreatment		
	Mean	SD	Mean	SD	*P*-value
VHI	12.55	2.56	18.26	3.72	<.001
Elasticity	2.70	0.57	3.95	0.71	<.001
Fluid volume	2.05	0.60	3.42	1.17	.001
pH	1.85	1.18	3.05	1.47	.014
Epithelial integrity	3.05	0.76	4.37	0.76	<.001
Moisture	2.90	1.33	3.53	1.12	.214

SD, standard deviation; VHI, Vaginal Health Index.

**Table 2.** Improvement in Parameters of Vulvar Health Index Pre- and Posttreatment

	Pretreatment		Posttreatment		
	Mean	SD	Mean	SD	*P*-value
VuHI	9.80	2.40	3.37	3.67	<.001
Labia	1.95	0.83	0.63	0.76	<.001
Clitoris	1.65	0.67	0.74	0.93	.004
Elasticity	1.55	0.83	0.42	0.61	<.001
Color	1.85	0.99	0.68	1.06	.002
Pain	2.80	0.52	0.89	0.99	<.001

SD, standard deviation; VuHI, Vulvar Health Index.

FSFI-6 scores showed a marked improvement from a baseline mean of 12.55 ± 4.70 to 17.95 ± 7.61 after treatment (*P* = .017), suggesting a reduction in sexual dysfunction and GSM-related symptoms. There was a significant increase in desire, satisfaction, and pain reduction, without a significant change in arousal, lubrication and orgasm, but clinical change was observed in the lubrication with an initial mean value of 1.70 ± 1.26 vs 2.68 ± 1.73 after treatment (Table 3).

**Table 3.** Improvement in Parameters of Female Sexual Function Index-6 Pre- and Posttreatment

	Pretreatment		Posttreatment		
	Mean	SD	Mean	SD	*P*-value
FSFI-6	12.55	4.70	17.95	7.61	.017
Desire	1.75	0.79	3.00	1.00	.001
Arousal	2.75	1.07	3.00	1.41	.560
Lubrication	1.70	1.26	2.68	1.73	.068
Orgasm	2.90	1.71	3.16	1.89	.720
Satisfaction	2.30	1.17	3.26	1.59	.041
Pain	1.15	0.59	2.84	1.80	.002

FSFI-6, Female Sexual Function Index-6; SD, standard deviation.

Pain levels related to the procedure remained low, with an average VAS score of 1.95 ± 0.94, with a maximum pain reported of 3, suggesting that the procedure was well tolerated.

Accordingly, when analyzing the patient-reported symptoms, dryness (with a mean value of 8.20 ± 1.58) and dyspareunia (9.10 ± 1.74) were considered by patients the causes of greater discomfort related to GSM. Both symptoms showed substantial improvements of 4.05 ± 3.52 (*P* = .001) and 0.15 ± 0.49 (*P* < .001) after the treatment, respectively (Table 4).

**Table 4.** Changes of Patient-Reported Symptoms Pre- and Posttreatment

	Pretreatment		Posttreatment		
	Mean	SD	Mean	SD	*P*-value
Dryness	8.20	1.58	4.05	3.52	.001
Itching when cleaning	2.40	3.14	0.15	0.67	.009
Dysuria	1.20	2.42	3.65	3.67	.042
Urinary urgency	1.15	2.39	0.15	0.67	.042
Pruritus	2.60	3.45	0.15	0.67	.008
Dyspareunia	9.10	1.74	0.15	0.49	<.001
Trigger point pain (6 o'clock)	1.75	3.49	0.05	0.22	.027

SD, standard deviation.

**Figure 2.**
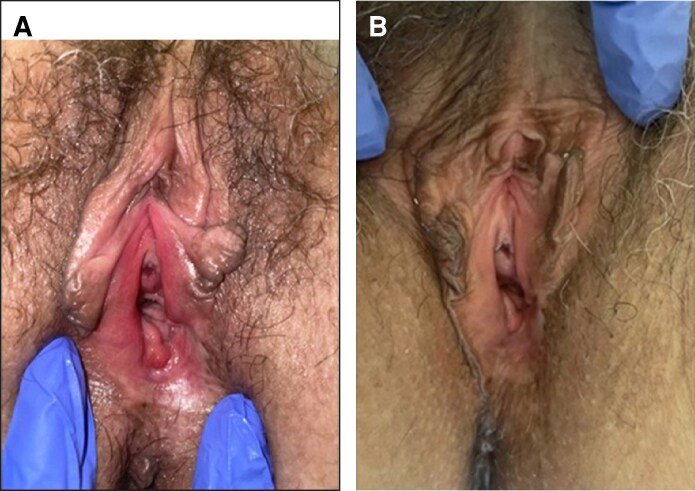
Representative clinical photographs of a 58-year-old woman (menopause since age 53) treated with carboxytherapy. Multiple infiltration sites were used (labia minora, vaginal introitus, lower third of vaginal mucosa, and clitoral hood) until achieving a self-resolving subcutaneous emphysema. Photographs were taken at (A) baseline and (B) 1 month after completion of treatment, showing improved mucosal coloration and hydration.

**Figure 3.**
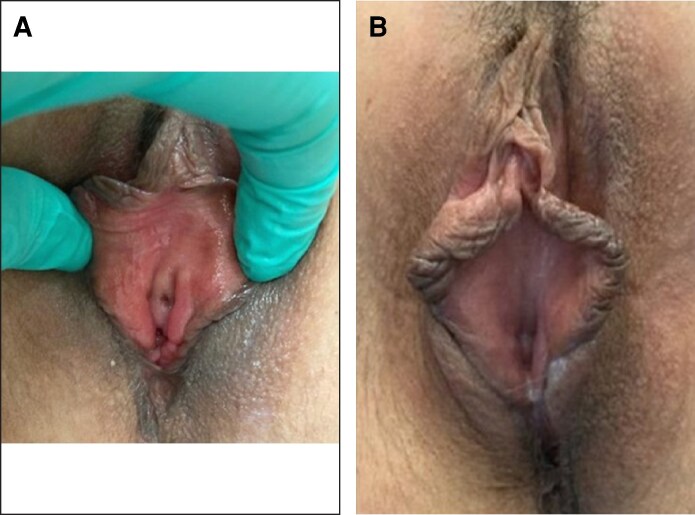
Representative clinical photographs of a 53-year-old woman (menopause since age 50) treated with carboxytherapy. Multiple infiltration sites were used (labia minora, vaginal introitus, lower third of vaginal mucosa, and clitoral hood) until achieving a self-resolving subcutaneous emphysema. Photographs were taken at (A) baseline and (B) 1 month after completion of treatment, showing improved mucosal coloration and hydration.

Visual inspection by gynecologists revealed improvements in vulvar appearance, with 13 patients showing substantial enhancement, 4 moderate improvements, and 1 mild change (representative clinical photographs are shown in Figures 2, 3).

No complications or adverse events were reported during the course of this study.

## DISCUSSION

In this prospective study, we found that patients with GSM who received carboxytherapy significantly improved vulvovaginal health scores, as well as sexual function, without associating significant side effects and with a good tolerance to the treatment.

These findings highlight the efficacy of carboxytherapy as a nonhormonal treatment for GSM, particularly in patients with significant vulvar involvement where conventional therapies may be less effective. Carboxytherapy's mechanisms enhancing oxygen delivery, inducing remodeling of the extracellular matrix, and promoting neoangiogenesis address the underlying tissue changes associated with GSM.^7,8,12^

Similar to studies on CO_2_ laser treatments for GSM, carboxytherapy demonstrated a high safety profile and patient tolerance, offering a potential alternative for individuals contraindicated for estrogen treatments.^12,13^ The combination of prewarmed CO_2_ at low flow rates and topical anesthesia likely contributed to the good tolerability observed in our cohort. Although the FSFI-6 score increased significantly after treatment, the mean value (17.9) remained below the cutoff of 19 that defines normal sexual function. This suggests that carboxytherapy led to improvement but did not fully restore sexual function. Clinically, it is recommended to first address primary symptoms such as dyspareunia, which often lead to sexual avoidance and reduced desire. To achieve a more complete recovery of sexual function, complementary strategies including sexual counseling tailored to the menopausal stage may be required.

Local treatments aiming at alleviating the symptoms of GSM are usually not well accepted by women. In cases with mild symptoms, it is recommended to start the treatment with lubricants, moisturizers, and the use of low-dose vaginal estrogens, which significantly reduce the most bothersome symptoms, including dyspareunia.^6,21^ However, a study of Spanish women found dissatisfaction with moisturizers by 28% related to their impact on sexual spontaneity, and estrogen users by 20% because of the time it takes to act.^17^ The use of regenerative techniques represents a new opportunity for the regeneration and restoration of vaginal health in menopausal women.

It is important to distinguish between basal vaginal mucosal hydration and lubrication during sexual intercourse. Hydration, reflected in measurements such as VHI, occurs continuously because of the adhesion of water (endogenous or applied) to the mucosa and contributes to tissue elasticity and health.^22^ On the other hand, sexual lubrication, a response triggered by arousal, results from vasocongestion and plasma transudation (arousal fluid), and constitutes a distinct physiological response not necessarily directly correlated with basal vaginal hydration.^23^

Carboxytherapy is credited with the ability to stimulate collagen renewal and improve cutaneous microcirculation by increasing blood flow.^24^ It also induces angiogenesis through both hypoxia-inducible factor-dependent and -independent mechanisms, promoting the formation of new blood vessels and improving the supply of oxygen to tissues. In addition, carboxytherapy can suppress inflammatory signaling, reduce oxidative stress, and blocks the production of proinflammatory cytokines, thereby limiting oxidative damage generated during inflammation.^25^

Despite these encouraging results, limitations of this pilot study include the small sample size, the lack of a control group, limited follow-up time, and restricted generalizability of the findings. A limitation of this study is that the photographic assessments were based on clinical judgment without the use of a standardized scoring scale, blinding, or inter-rater comparison. Another limitation is that patient-reported scales were completed in a nonanonymous setting, which may have introduced a response bias. Although the distribution of CO_2_ and the number of injection sites varied according to individual anatomy and tissue response, this is inherent to the technique and represents a personalized approach rather than a methodological limitation. Future studies should use randomized, with comparator groups, comparator-controlled designs with larger samples to validate these preliminary findings. Additional investigations should also examine the long-term benefits of carboxytherapy in maintaining tissue health and reducing recurrent GSM symptoms.

## CONCLUSIONS

Carboxytherapy appears to be a safe, effective, and well-tolerated treatment option for GSM, providing symptomatic relief, enhancing both vulvar and vaginal health markers and improving sexual experiences of women. Larger, controlled studies are needed to confirm these findings and optimize carboxytherapy protocols for GSM management.
